# An Evaluation of Age-Group Latent Mean Differences in Maladaptive Identity in Adolescence

**DOI:** 10.3389/fpsyt.2021.730415

**Published:** 2021-09-17

**Authors:** Carla Sharp, Salome Vanwoerden, Klaus Schmeck, Marc Birkhölzer, Kirstin Goth

**Affiliations:** ^1^Department of Psychology, University of Houston, Houston, TX, United States; ^2^Department of Child and Adolescent Psychiatry, University Clinics of Basel, Basel, Switzerland

**Keywords:** level of personality function, AMPD Criterion A, personality disorder, adolescence, maladaptive identity

## Abstract

Little is known about the differences between age groups in maladaptive personality function as denoted in Criterion A of the Alternative Model for Personality Disorder (AMPD) in the DSM-5, which is the entry criterion for diagnosing personality disorder in the upcoming ICD-11. The current study aimed to address this gap by evaluating latent mean age group differences in maladaptive identity, which is one aspect that has been identified as an important feature of maladaptive, general personality function as represented in the DSM-5 and ICD-11. We were also interested whether mean differences would track with mean differences in borderline personality disorder (BPD) features given prior data suggesting that general personality function overlap with the construct of BPD. A community sample of *N* = 2,381 adolescents, representing a mix of different socio-economic and educational backgrounds, ages 12-18 (*M* = 14.92, *SD* = 1.94; 46% male) completed a measure of maladaptive identity. A subset (*n* = 1,165) completed a measure of borderline personality features. Latent variable modeling was used to evaluate latent mean differences across seven age bands. Results suggested a normative increase in maladaptive identity after age 12, which remained consistent until age 17 when it dropped back to levels observed in 12-year-olds. Maladaptive identity was significantly associated with mean-level increases in borderline personality features, with these constructs becoming more closely associated with increasing age.

## Introduction

The publication of the Alternative Model for Personality Disorders [AMPD; (1)] introduces maladaptive self- and inter-personal functioning (Criterion A) as a unidimensional severity continuum common (general) and core to all personality pathology. Criterion A is referred to as the Level of Personality Functioning (LPF) and includes disturbances in identity and self-direction (self-function) and intimacy and empathy (interpersonal function). Once a clinician has determined a client's LPF, she/he then determines the client's level of maladaptive personality trait function (Criterion B) across five trait domains (negative affectivity, detachment, antagonism, disinhibition, and psychoticism), which encompass 25 trait facets. Similarly, the 11th revision of the International Classification of Diseases [ICD-11; (2)] adopted a dimensional approach to the classification of personality disorders with its entry criterion defined as impaired self- and inter-personal functioning, followed by evaluation of five trait qualifiers (3, 4).

Interest in the developmental aspects of personality disorder has increased over the last two decades, motivated by research showing that personality disorder onsets in adolescence; therefore, early identification and intervention in adolescence may prevent significant suffering and cost for individuals and families (5–7). While a robust literature exists supporting both the traditional DSM-5 section II conceptualization of borderline personality disorder in adolescence, and that of DSM-5 Section III Criterion B/ICD-11 maladaptive traits in children and adolescents, much less research has been conducted on the entry criteria of both the AMPD and the ICD-11 formulations (maladaptive self- and inter-personal function) in adolescents (8). Hence, little is known about the mean differences between age groups in the common features of maladaptive personality function. Knowing whether to expect increases or decreases in maladaptive self and interpersonal function as young people develop through adolescence would help set expectations so that deviations from typical patterns can be identified (and treated, if necessary). Research on age mean differences in borderline traits as well Criterion B maladaptive trait function has typically shown higher means of maladaptive personality traits in mid- and late-adolescence compared to pre- or early-adolescence and young adulthood [e.g., (9, 10)]. As yet, it is not known whether mean differences among different age groups in maladaptive self and interpersonal function would follow a similar pattern.

Against this background, the current study aimed, first, to evaluate latent mean age differences across adolescence in one aspect of general personality function, namely maladaptive self and identity function. In both the AMPD and the ICD-11 formulations, maladaptive self and identity function forms a key component of the entry criterion of personality disorder. In the AMPD, for example, severe manifestations for disturbances in self and identity function include problems in experiencing oneself as unique with a sense of agency or autonomy, boundary problems, an incoherent self-image, fragile self-esteem, poor self-regulation, difficulties in establishing or achieving personal goals, and compromised ability to reflect on and understand own mental processes. Similarly, the ICD-11 operationalizes identity disturbance through stability and coherence of one's sense of identity, ability to maintain an overall positive and stable sense of self-worth, accuracy of one's view of one's characteristics, strengths, limitations and the capacity for self-direction (ability to plan, choose, and implement appropriate goals). Self and identity function is therefore increasingly recognized as a central dimension of personality pathology in both adults and adolescents (11, 12).

Our central hypothesis was that children in mid-adolescence would show higher levels of maladaptive self and identity function compared to early and late adolescents. This is based, firstly, on the Erikson's (13) theory of identity crisis in mid-adolescence; and the findings consistent with this theory from studies of adaptive self- and identity-development showing that as adolescents age into young adulthood, they progress through an identity formation process from an identity based on identifications (foreclosure status), through an exploration (moratorium) process, to a new configuration, based on the sum of its identificatory elements (achievement) (14, 15). Therefore, mid-adolescence tends to be associated with a period of increased identity diffusion associated with exploration until adolescents reach a more consolidated sense of self toward the end of adolescence and early adulthood. Second, there is strong correlation between Criterion A and B traits [see (16) for a review of this literature]. If Criterion B maladaptive traits have higher means in mid adolescence than early and late adolescence as discussed earlier, it follows that maladaptive Criterion A function may also evidence the same pattern given high correlations between Criterion A and B.

To evaluate our hypotheses, we used latent variable modeling to evaluate age invariance in a large community-based sample of adolescents across seven age bands. Latent variable modeling (as opposed to mean difference scores in observed scores) offers a robust approach to evaluating age-group differences (17), because it models latent means that take into account measurement error that may bias estimates of the relations among the underlying constructs [e.g., (18)], thereby allowing for inference of valid comparisons across groups or over time (19).

As a second aim, we investigated associations between maladaptive identity and borderline features across different age groups to answer the question whether maladaptive identity tracks with adolescent personality pathology, as defined in Section II of the DSM-5. This is an important question for two reasons. The first relates to the suggested notion that borderline features, as traditionally defined in Section II of the DSM-5, reflects the general, shared features of personality pathology in the same way that Criterion A does (16, 20, 21). Demonstrating similar age mean differences in the two constructs simultaneously would offer further support that these two constructs are inextricably linked. Second, demonstrating associations of latent age mean differences between maladaptive identity and borderline features would further validate the relevance of maladaptive identity for personality pathology. Given well-established cross-sectional findings showing identity disturbance to be associated with borderline personality disorder features in adults and adolescents (22), combined with evidence of increases in both maladaptive traits and borderline features in mid-to-late adolescence discussed earlier, we expected that higher levels of maladaptive identity associated with mid-adolescence would track with borderline personality disorder features.

## Methods

### Participants

A sample of *N* = 2,381 adolescents were recruited from the community in Germany and Switzerland at 11 schools, representing a mix of different socio-economic and educational backgrounds, aged 12-18 (*M* = 14.92, *SD* = 1.94; 46% male). The schools were selected to be representative and included junior high schools, middle schools, high schools, and vocational schools from urban and rural areas. There were roughly equal numbers of adolescents in each age group, which are detailed in **Table 2**. Data collection took place at the schools in a group-setting by classes or grades during one school hour. The adolescents were asked to fill out the questionnaires without talking, supervised by an undergraduate research assistant who was available to answer questions. Prior to the assessment, the study was explained to the students and parents gave a written informed consent for study participation. A subset (*n* = 1,165) completed a measure of borderline personality features (BPFSC-11).

### Measures

The Assessment of Identity Development in Adolescents [AIDA; (23)] assesses impairments in self and identity functioning, we use the AIDA, a measure specifically developed to capture maladaptive self and identity function in adolescents, purported to be a core dimension of personality pathology according to DSM-5 Section III (1, 13, 24), OPD-CA2 and the upcoming ICD-11 (2). The AIDA is a 58-item self-report measure for adolescents aged 12-18 years with five-option answering format (0 = no to 4 = yes). All items add up to the total scale Identity Diffusion; high scores suggest a high level of impairment. Similar to measures of AMPD informed maladaptive self- and identity-function, the AIDA therefore probes both adaptive or typical self- and identity-function (e.g., individuals can score 0 on all or most items) and maladaptive identity function. It therefore deviates from prior measures of maladaptive identity function in psychopathology research, which typically focus only on extreme ends of the severity continuum, as well as prior measures of adaptive identity functioning that do not provide adequate coverage of maladaptive identity function (25).

The construction of the AIDA was top-down and focused on clinical validity by integrating those aspects of self and identity development from different schools of thought that had empirically shown to be clinically valid in the description of relevant impairments; i.e., that had the potential to significantly discriminate healthy persons from personality disordered persons (25). Thus, to provide adequate coverage of the full construct of identity pathology, six different relevant aspects of impairments were combined to build the full AIDA-model and ordered into the two primary scales “Continuity” and “Coherence” that are also used in the OPD-CA2. For each aspect, item formulations had been developed that are short, unambiguous, clearly representing a variation from “healthy-to-impaired,” and easy to understand. For example for the area “Continuity,” aspect “identity-consolidating perspectives” the item: “I could list a few things that I can do very well.” (reverse scoring) or for the aspect “identity-consolidating roles” the item: “I feel like I m a valuable member of my family.” (reverse scoring). Likewise for the area “Consistency,” aspect “identity-integrating consistency in self-concepts” the item: “I often feel lost, as if I had no clear inner self.” Or for the aspect “identity-integrating cognitive self-experience” the item: “I am confused about what kind of person I really am.”

All items underwent empirical beta and pilot and validation tests before being integrated into the questionnaire. In order to be transparent concerning the roots and the full scope of the concept and in order to enable the investigation of possible distinct relations of the domain constructs (e.g., concerning relations to external variables, therapeutic focus or prognostic outcomes) the domain constructs may be used as subscales and scales in terms of narrative descriptive units. Subscales are not supposed to be statistically independent scales but on the contrary, they are supposed to represent the clinical complex, but joint factor “Self and Identity pathology” together. Exploratory factor analysis supported a one-factor solution supporting a joint factor (23). However, scale reliabilities were good with Cronbach's alpha 0.94 on total, 0.87 and 0.92 on primary and 0.69 to 0.84 also on the subscale level and support the possibility of using the subscales as descriptive units.

Most important, the AIDA has shown excellent clinical utility. The AIDA total score of Identity Diffusion differed at a highly significant level and with a relevant effect size of *d* = 2.6 standard deviations between the general population (Mean 64.9, SD 27.6) and patients diagnosed with BPD according the *SCID-2 (*Mean 137.6, SD 25.1). The difference with patients with other PD types reached an effect size of *d* = 2.0, and patients without any PD (patients with internalizing or externalizing disorders) of *d* = 0.9. This speaks to the high relevance of the construct assessed by the AIDA to describe impairments associated with especially BPD but also other PD pathology (25, 26).

The AIDA was initially constructed in the German language. The development of versions in other languages includes culture-adapted translation by experts in the field of child and adolescent psychology, back-translation process and discussion with the original authors and empirical pilot and main tests in school and clinical samples to ensure basic psychometric qualities. Several translated versions had shown excellent internal consistency and construct validity, e.g., among Spanish speaking adolescents in Mexico (27) and English speaking adolescents in the US (28). In the current sample, internal consistency was excellent for the total score (α = 0.94).

The Borderline Personality Features Scale for Children [BPFS-C-11; (29)] is an 11-item self-report measure of borderline personality features for children 9-18 years old. The BPFS-C-11 was developed using item response theory of the full, 24-item version of the measure (30) and has since been validated in separate samples demonstrating good criterion validity, internal consistency, and test-retest reliability (29, 31) and gender invariance (32). The German version of the BPFS-C-11 was developed using typical translation and back-translation procedures and evaluated in a pilot validation sample of n = 393 adolescents. In the current sample, Cronbach's α was 0.82.

### Data Analytic Strategy

Descriptive analyses were conducted using SPSS 25 (33), factor analyses were conducted using Mplus 7 (34), and TVEM analyses were conducted using SAS 9.4 (35). There were four cases with missing data on a single scale; specifically, one case was missing the scale of Incoherence—Cognition; two cases missing Incoherence—Autonomy; and one case missing Discontinuity—Attributes. This amount of missing data was minimal and was estimated using used maximum likelihood (ML) estimation. Missing data was minimal with no more than two cases with missing data. Values of skew and kurtosis ranged from 0.02 to 1.55 indicating that distribution of scales approximated a normal distribution in the full sample (see Table 1). Bivariate correlations were examined within the full sample. Fit of each model was examined using multiple fit indices (18): the root mean square error of approximation (RMSEA), with values of <0.08 indicating reasonable fit and values above 0.10 suggesting poor fit (36); the comparative fit index [CFI; (37)], with values between 0.95 and 1.00 indicating excellent fit and values between 0.90 and 0.95 indicating acceptable fit (38); and the standardized root mean square residual (SRMR), with values <0.08 indicating acceptable fit (38).

**Table 1:** Descriptive statistics and correlations for AIDA scales in full sample (*N* = 2,381).

	**1**	**2**	**3**	**4**	**5**	**6**
1. Discontinuity—attributes						
2. Discontinuity—relationships	0.52^**^					
3. Discontinuity—emotions	0.46^**^	0.63^**^				
4. Incoherence—self-consistency	0.49^**^	0.70^**^	0.73^**^			
5. Incoherence—autonomy	0.37^**^	0.54^**^	0.67^**^	0.65^**^		
6. Incoherence—cognitions	0.43^**^	0.60^**^	0.69^**^	0.72^**^	0.67^**^	
7. Borderline features	0.44^**^	0.62^**^	0.74^**^	0.75^**^	0.65^**^	0.70^**^
Mean (SD)	13.16 (5.51)	7.93 (6.10)	8.47 (5.06)	13.76 (7.93)	15.48 (7.47)	10.54 (5.45)
Range	0-36	0-40	0-28	0-44	0-48	0-32
Skew	0.37	1.15	0.67	0.71	0.48	0.46
Kurtosis	0.32	1.55	0.25	0.20	0.30	0.02

** *p < 0.01*.

Prior to measurement invariance analyses, confirmatory factor analysis was used to evaluate model fit of a single factor defined by the six subscales within the full sample. Next, measurement invariance was examined across seven age groups using a hierarchical set of multigroup CFAs, with each subsequent model imposing additional constraints of equality across age groups. The baseline model tested configural invariance to examine whether the single factor structure of maladaptive identity was invariant across age groups. Next, metric invariance was tested to evaluate whether the pattern of factor loadings were equal across age groups. Finally, scalar invariance was tested to evaluate whether item intercepts were equal across age groups. Considering χ^2^ difference tests are susceptible to similar problems as the χ^2^, including sample size dependency (18), two separate fit indices were used to evaluate difference in model fit: CFI change of <0.010 and RMSEA change of <0.015 (39) provided statistical evidence for invariance between the less constrained and more constrained model. Following invariance testing, latent factor means for identity diffusion adjusted for any invariance found were compared across age groups (each year compared to age 12 and the previous age group).

The association between borderline personality features and identity disturbance change over the course of adolescence was tested using the time-varying effect model [TVEM; (40, 41)], which estimates regression coefficients as a function of age. Intercept-only TVEM was used to examine borderline personality features across the age groups included in the study as a function of identity disturbance. Resulting regression coefficients are age-varying coefficients that expresses the change in borderline personality features for each unit change in identity disturbance as a smooth, non-parametric function of age. These models were run in SAS 9.4 using the %normal_TVEM macro [TVEM; (42)]. P-spline smoothing was used for the model, which automatically selects the optimal form of each coefficient function. These results are presented as a figure (**Figure 2**) because coefficients are estimated as a function of continuous time, creating a number of coefficients across age too large to be presented in the text or a table.

## Results

### Descriptive Results

Given that a subset of adolescents completed the BPFSC-11, we first examined differences between BPFSC-11 completers and non-completers. These results showed that completers were older (*M* = 15.10, *SD* = 1.90; *t*(2378.88) = −4.26, *p* < 0.001; *d* = 0.18), scored higher (more maladaptive) on the AIDA (*M* = 73.58, *SD* = 33.14; *t*(2259.10) = −6.64, *p* < 0.001; *d* = 0.27), and were 1.41 times more likely to be male [50% male; $\chi_{\left(1\right)}^{2}$ = 17.42, *p* < 0.001] compared to non-completers (*M*_*age*_ = 14.76, *SD* = 1.97; *M*_*LOPF*_ = 65.30, *SD* = 27.39; 42% male). While these differences were statistically significant, effect sizes were small to minimal.

Table 1 lists the descriptive statistics of the six AIDA subscales within the full sample and Table 2 describes means and standard deviations of the subscales and total score within each age group.

**Table 2:** Descriptive statistics by age group for observed and latent variables of identity diffusion.

	**Age 12**	**Age 13**	**Age 14**	**Age 15**	**Age 16**	**Age 17**	**Age 18**
*N*	330	346	378	350	362	329	286
**Observed**							
Identity diffusion	64.04 (30.56)	61.81 (99.21)	68.02 (60.95)	74.61 (31.79)	73.09 (28.10)	61.17 (28.84)	63.51 (28.81)
Discontinuity—attributes	12.69 (5.28)	13.45 (5.60)	13.65 (5.75)	14.07 (5.72)	13.60 (5.16)	12.49 (5.50)	11.81 (5.22)
Discontinuity—relationships	6.73 (5.61)	8.11 (6.48)	7.83 (6.44)	9.14 (6.81)	8.71 (5.69)	7.54 (5.56)	7.24 (5.56)
Discontinuity—emotions	8.19 (5.42)	9.08 (5.52)	8.71 (5.21)	9.06 (5.06)	8.97 (4.73)	7.89 (4.72)	7.06 (4.22)
Incoherence—self-consistency	12.40 (7.96)	13.66 (8.46)	13.89 (8.23)	14.97 (8.29)	14.73 (7.49)	13.90 (7.70)	12.40 (6.71)
Incoherence—autonomy	14.45 (7.80)	15.64 (8.14)	15.62 (7.94)	16.06 (7.67)	16.22 (6.75)	15.21 (7.04)	14.93 (6.49)
Incoherence—cognitions	9.58 (5.83)	10.65 (6.02)	11.00 (5.67)	11.32 (5.54)	10.84 (4.93)	10.13 (4.93)	10.08 (4.88)
Latent							
Identity diffusion	0.00	0.18 (0.08)^*^	0.20 (0.08)^*^	0.33 (0.08)^**^	0.32 (0.09)^**^	0.11 (0.09)	−0.05 (0.09)

* *p < 0.05*,

** *p < 0.01; descriptive statistics of observed variables include M and SD whereas descriptive statistics of latent variables include M and SE*.

Bivariate correlations revealed that subscales were all interrelated to a moderate to strong degree. AIDA subscales and borderline features correlated positively and strongly.

### Age Invariance Results

Before conducting invariance testing, a confirmatory factor analysis was conducted; the model specified a single latent variable of maladaptive identity defined by the six AIDA subscales. The model was identified by fixing the factor variance to one and freely estimating all factor loadings. No covariances between subscales were estimated. This model demonstrated good fit to the data [$\chi_{\left(9\right)}^{2}$ = 206.04, *p* < 0.001; RMSEA = 0.096; CFI = 0.976; SRMR = 0.027] and standardized estimates of factor loadings ranged from 0.554 (Discontinuity in attributes and goals) to 0.876 (Incoherence in consistent self-image) suggesting that a single dimension of maladaptive identity adequately represented variability across the different subscales.

Measurement invariance of this single factor model was tested across the seven age groups. First, to test configural invariance, the single factor model was evaluated across all age groups with factor loadings free to vary across groups. This model had satisfactory fit only across two out of three indices [χ^2^_(63)_ = 264.72, *p* < 0.001; RMSEA = 0.097; CFI = 0.976; SRMR = 0.031]. Modification indices were examined to determine what model changes may improve fit; it was suggested to allow Discontinuity in attributes and goals to correlate with Discontinuity in relationships and roles among 14-year-olds; however, RMSEA of this model was still not in the satisfactory range. The next model allowed Incoherence in consistent self-image to correlate with Discontinuity in relationships and roles among 12-year-olds; this model demonstrated satisfactory fit to the data [χ^2^_(61)_ = 186.296, *p* < 0.001; RMSEA = 0.078; CFI = 0.985; SRMR = 0.025], thereby making it the baseline model from which subsequent models were compared to.

To evaluate metric invariance, factor loadings were constrained to be equal across all age groups and the factor variance for all non-reference groups was freely estimated. This model demonstrated good fit [χ^2^_(91)_ = 267.839, *p* < 0.001; RMSEA = 0.076; CFI = 0.979; SRMR = 0.058] and change in CFI and RMSEA was below stated limits suggesting that there was metric invariance for the single factor model of maladaptive identity.

Finally, scalar invariance was tested by constraining intercepts to be equivalent across groups and allowing the factor mean for all non-reference groups to be freely estimated. This model demonstrated good fit [χ^2^_(121)_ = 370.571, *p* < 0.001; RMSEA = 0.078; CFI = 0.970; SRMR = 0.065] and change in CFI and RMSEA was below stated limits suggesting that there was scalar invariance for the single factor model of maladaptive identity.

### Aim 1: Latent Mean Differences in Maladaptive Identity Across Age Groups

**Figure 1:**
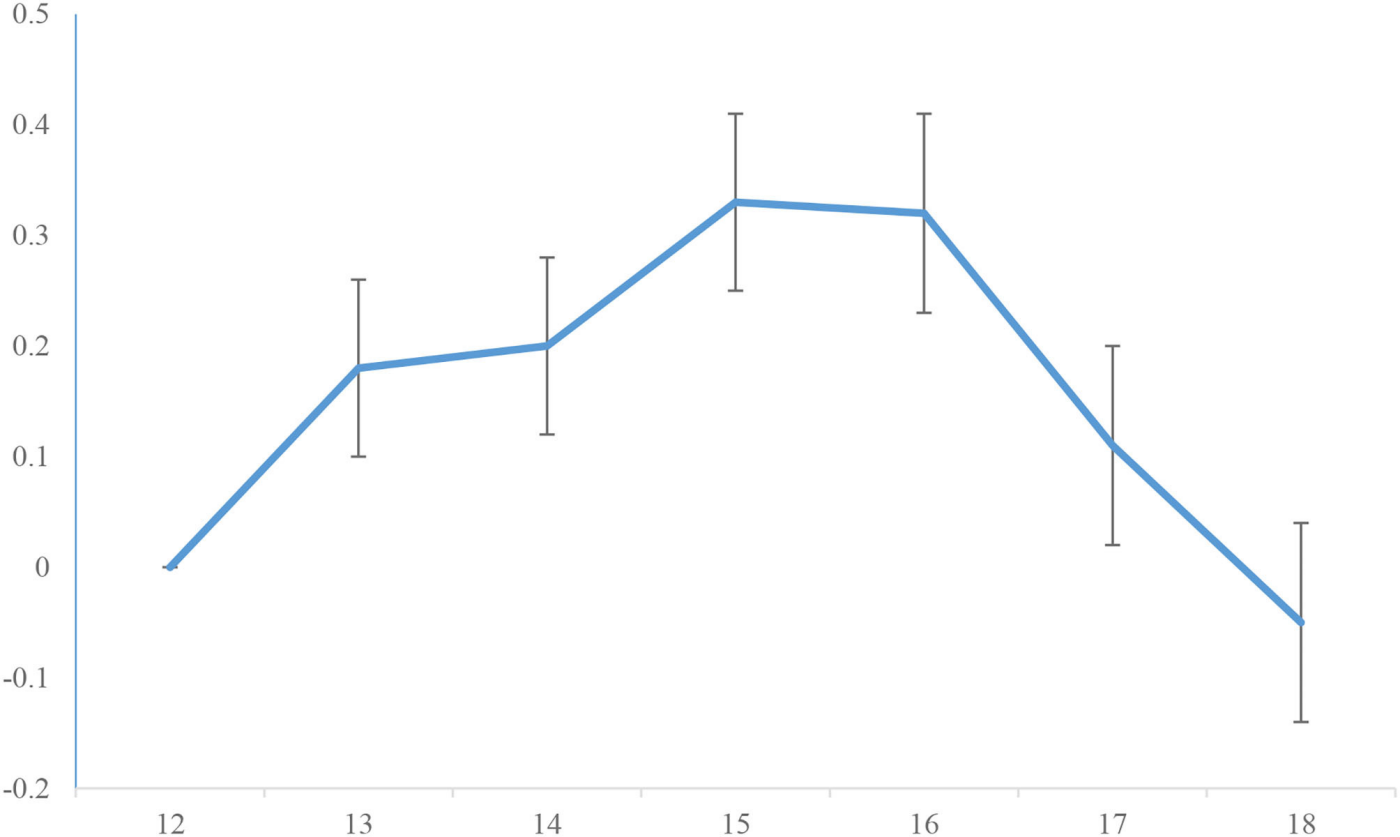
Plot of latent means of identity diffusion for age groups 12 through 18.

Because the latent mean of maladaptive identity was set to zero among 12-year-olds for identification purposes in the scalar model, latent means in subsequent age groups (listed in Table 2) could be examined for significant change from 12-year-olds. Results indicated that levels of maladaptive identity in 13-16-year-olds were significantly higher than latent mean levels in 12-year-olds. Latent means among 17- and 18-year olds were not significantly different from 12-year-olds. Comparisons between adjacent age groups using Wald tests demonstrated that there was no significant increase in maladaptive identity between 13- and 14-year olds [0.01(0.08), *p* = 0.929], 14- and 15-year olds [0.12(0.08), *p* = 0.122], 15- and 16-year olds [−0.05(0.08), *p* = 0.540], 17- and 18-year olds [−0.14(0.07), *p* = 0.068]. However, there was a significant decrease in maladaptive identity between 16- and 17-year olds [−0.19(0.07), *p* = 0.010]. These findings are visually represented in Figure 1.

### Aim 2: Associations Between Identity Disturbance and Borderline Features Across Age Groups

**Figure 2:**
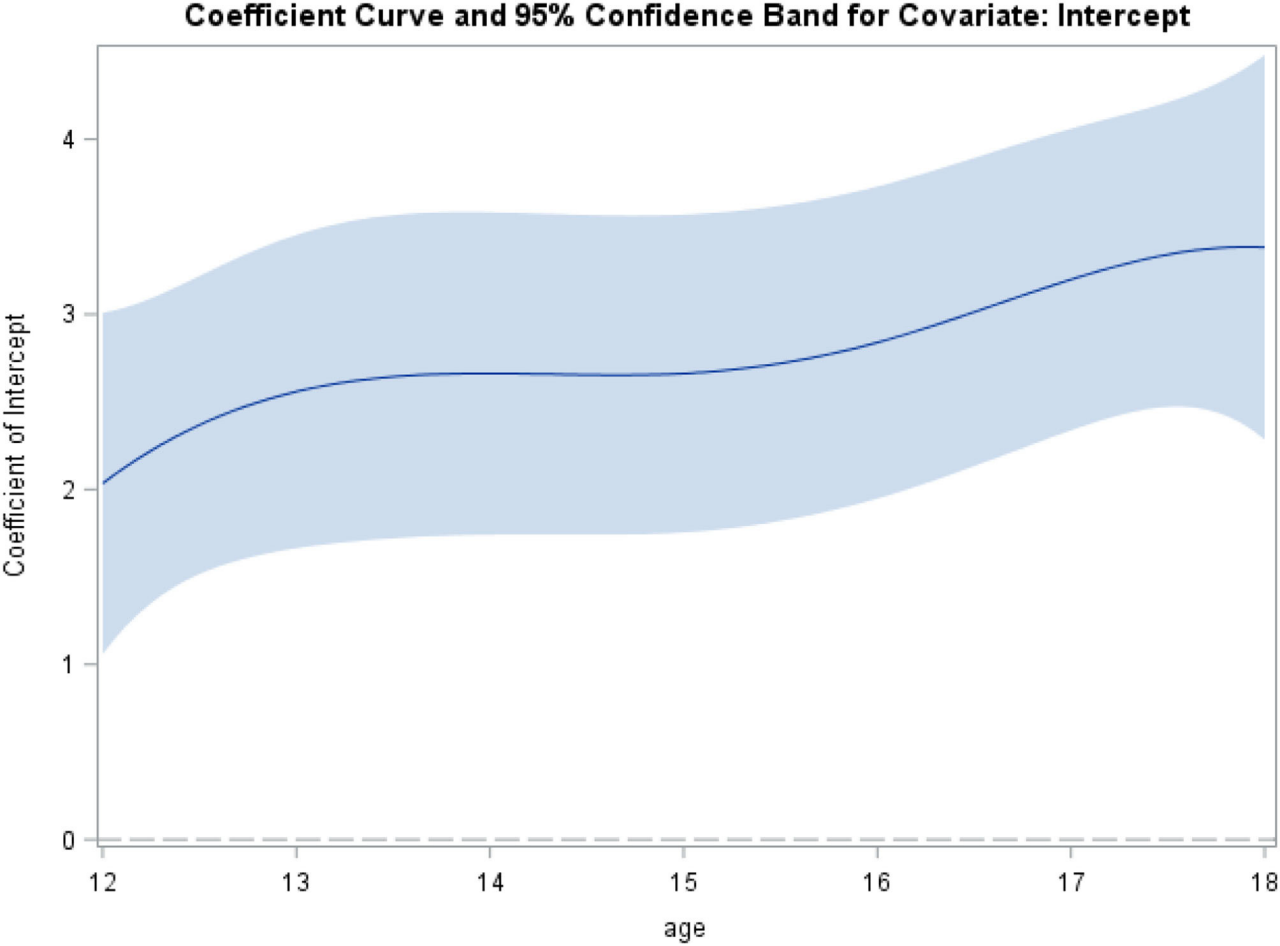
Age-varying associations between borderline personality features and identity disturbance.

Lastly, we examined age-varying associations between borderline personality features and identity disturbance. Figure 2 presents the TVEM estimates plotted across age with 95% confidence intervals. We observed a positive and increasing association with borderline personality features between ages 12 to 13 (estimated value_age12_ = 1.92, SE = 0.48; 95% CI: 0.98, 2.86; estimated value_age13_ = 2.44, SE = 0.42; 95% CI: 1.62, 3.26), which largely leveled off, and then increased again from ages 15 to 18 (estimated value_age15_ = 2.51, SE = 0.42; 95% CI: 1.70, 3.33. The strongest association was observed around the age of 18 (estimated value = 3.26, SE = 0.54; 95% CI: 2.20, 4.31). These results suggest that throughout adolescence, maladaptive identity is significantly associated with increased borderline personality features, with these constructs becoming more closely associated with increasing age.

## Discussion

An empirically-based understanding of mean age differences in maladaptive personality is important to identify correlates and predictors of deviation from typical development in service of the early identification and treatment of personality pathology in young people (5–7). The current study aimed to evaluate age-group latent mean differences for maladaptive identity, which is one aspect of Level of Personality Functioning as well as ICD-11 entry criterion, in a large community sample of adolescents. We were also interested in whether latent mean differences would track with latent mean differences in borderline pathology as conceptualized in Section II of the DSM-5.

Regarding our first aim, and consistent with our central hypothesis, our results suggest similar age mean differences for maladaptive identity development previously shown for maladaptive trait development [e.g., (43)], and DSM-5 Section II borderline personality disorder [e.g., (9, 21)]. Specifically, our results suggest higher levels of maladaptive identity after age 12, which remains consistent until age 17, after which it drops down to mean levels observed in 12-year-olds. Regarding our second aim, we showed that maladaptive identity is significantly associated with mean-level increases in borderline personality features, with these constructs becoming more closely associated with increasing age. This finding was consistent with our expectations based on prior cross-sectional research that have established an association between identity disturbance and borderline personality disorder (22). It was also consistent with more recent work utilizing another new measure of AMPD informed maladaptive identity function, the Dutch version of the Self-Concept and Identity Measure [SCIM; (44, 45)]. While this study did not examine associations between mean-level increases over time, it did show correlations between the SCIM and the BPFSC-11, as used in the current study.

Scholars from diverse theoretical backgrounds converge on the notion that a well-functioning identity enables one to experience feelings of personal meaning and well-being and to find satisfying and fulfilling engagement in one's social context (46–50). These scholars also converge on the idea that adolescence confer a critical developmental period for the formation of a healthy identity (14). Substantial developmental research has been conducted to document progressive movement through Erikson's (47) identity formation process, from an identity based on identifications (foreclosure status), through an exploration (moratorium) process, to a new configuration, based on, but different from, the sum of its identificatory elements (achievement) (14, 15). By showing a return to baseline in maladaptive identity function by age 17, coupled with a strengthening of the association between maladaptive identity function and borderline features with increased age, our findings suggest an expected trajectory for the normative increase in maladaptive personality that can serve as a benchmark against which deviations can be monitored. While prominent scholars in the field have suggested that this be done (51, 52), until now, an empirically established expectation for age related changes in maladaptive personality function has not yet been determined. Pending replication, and given the nature of our sample—a community-based sample—our study provides the first description of a potential trajectory expected for typical development of Criterion A informed identity function against which atypical patterns can be evaluated. Clinical use of this normative trajectory as benchmark would necessitate explicit norms which could guide decision-making on whether an adolescent's scores are elevated even if normative elevation is expected during middle adolescence. Adolescents with scores that significantly exceed the normative trajectory can thus be identified for intervention. Similarly, adolescents who stayed on the curve through middle adolescence, but failed to follow the normative decline in identity diffusion by late adolescence, can also be identified for intervention. By demonstrating strong correlations between means across different age groups between borderline features and maladaptive identity, the current study confirms that Criterion A (as measured by the AIDA) assesses a construct relevant to personality pathology in adolescence; and further emphasizes the need for intervention in adolescents who “fall off the normative curve.”

That maladaptive identity and personality pathology both increase over adolescence as demonstrated in the current study is consistent with developmental considerations incorporated in the ICD-11 (2) conceptualization of personality disorder. The ICD-11, for instance points out that “Personality Disorder is not typically diagnosed in pre-adolescent children. Over the course of their development, children integrate knowledge and experience about themselves and other people into a coherent identity and sense of self, as well as into individual styles of interacting with others. Different children vary substantially in the rate at which this integration occurs, and there is also substantial variation in the rate of integration within individuals over time. Therefore, it is very difficult to determine whether a pre-adolescent child exhibits problems in functioning in aspects of the self, such as identity, self-worth, accuracy of self-view, or self-direction, because these functions are not fully developed in children.” These ideas about how personality disorder is tied to self-and identity development has been the focus of recent work in developmental personality pathology (11, 12).

Our findings also contribute to the psychometric basis of the AIDA. First, the AIDA items appear to be best represented by a single latent factor of maladaptive self and identity function. This finding is consistent with prior studies using the AIDA (53), and is of note especially when considering the fact that the AIDA was developed, in accordance with most theories of identity (46–50) and identity diffusion (54, 55) to cover both intra- and interpersonal components (25). While the extent to which the AIDA overlaps with measures of Level of Personality Function remains an empirical question, factor analytic evidence for measures of the Level of Personality Function Scale suggest a unidimensional factor structure (56–58) consistent with the idea that aspects of self and interpersonal function are inextricably linked (59, 60).

Second, we demonstrated that the AIDA is equally valid for use across adolescent age groups. Invariance suggest that underlying factors really do reflect the same construct and that measurements themselves operate in the same way across age groups which is important in controversies of “changing persons vs. changing tests” (61). Even so, such methodological considerations are still regularly disregarded in contemporary applied developmental research (62). Here, we demonstrate that the AIDA taps the latent construct of maladaptive identity development similarly across adolescent age groups so that meaningful developmental inferences and comparisons can be made. Other studies have shown similar age invariance of measures of adaptive identity function based on Erikson's (13) model [e.g., (63)] as well as maladaptive identity function (64). This should facilitate further work in continuity and change also for within person development studies of maladaptive identity.

The current study has several limitations. First, while we sampled a considerable number of adolescents across ages, the cross-sectional nature of our study limits within-person developmental inferences that can be made. Future research should examine these constructs longitudinally and furthermore examine growth curves individually and in association with each other. Helpful examples in this regard can be drawn on from typical/adaptive identity development literature [e.g., (63)]. In addition, our sample was limited to Swiss and German adolescents and there is a need to replicate these findings among those from various ethno-cultural backgrounds, as well as clinical populations. Finally, follow-up through young adulthood would add important information about the expected age-related changes in maladaptive identity beyond adolescence.

Despite these limitations, we hope that these findings begin to provide preliminary support for the idea that adaptive self and identity function (which is intractably linked to adaptive interpersonal function) constitute a developmental milestone, that, if missed, may impede the binding of personality, and ultimately, the healthy transformation from child to adult personality function (12, 16).

## Data Availability Statement

The raw data supporting the conclusions of this article will be made available by the authors, without undue reservation.

## Ethics Statement

The studies involving human participants were reviewed and approved by Ethics Committee of Psychiatric University Clinics (UPK) Basel. Written informed consent to participate in this study was provided by the participants' legal guardian/next of kin.

## Author Contributions

CS conceptualized the study and analytic plan and wrote the first draft. SV conducted the analyses and made contributions to all aspects of the manuscript. MB, KS, and KG collected the data, were principal investigators on the study, and provided input on the methods, introduction, and discussion sections of the paper. All authors contributed to the article and approved the submitted version.

## Conflict of Interest

The authors declare that the research was conducted in the absence of any commercial or financial relationships that could be construed as a potential conflict of interest.

## Publisher's Note

All claims expressed in this article are solely those of the authors and do not necessarily represent those of their affiliated organizations, or those of the publisher, the editors and the reviewers. Any product that may be evaluated in this article, or claim that may be made by its manufacturer, is not guaranteed or endorsed by the publisher.
